# The Stranding Anomaly as Population Indicator: The Case of Harbour Porpoise *Phocoena phocoena* in North-Western Europe

**DOI:** 10.1371/journal.pone.0062180

**Published:** 2013-04-22

**Authors:** Helene Peltier, Hans J. Baagøe, Kees C. J. Camphuysen, Richard Czeck, Willy Dabin, Pierre Daniel, Rob Deaville, Jan Haelters, Thierry Jauniaux, Lasse F. Jensen, Paul D. Jepson, Guido O. Keijl, Ursula Siebert, Olivier Van Canneyt, Vincent Ridoux

**Affiliations:** 1 Laboratoire Littoral Environnement et Sociétés, UMR 7266, Université de La Rochelle, La Rochelle, France; 2 Observatoire *PELAGIS*, UMS 3462 - Université de La Rochelle-CNRS, Université de La Rochelle, La Rochelle, France; 3 Zoological Museum, The Natural History Museum of Denmark. University of Copenhagen, Copenhagen Ø, Denmark; 4 Marine Ecology Department, Royal Netherlands Institute for Sea Research (NIOZ), Texel, The Netherlands; 5 The Wadden Sea National Park Authority of Lower Saxony, Wilhelmshaven, Germany; 6 Météo France, Dprevi/MAR, Toulouse, France; 7 Cetacean Strandings Investigation Programme, Institute of Zoology, Zoological Society of London, London, United Kingdom; 8 Management Unit of the North Sea Mathematical Models (MUMM), Royal Belgian Institute of Natural Sciences (RBINS), Oostende, Belgium; 9 Department of Pathology, Veterinary College, University of Liege, Liege, Belgium; 10 Fisheries and Maritime Museum, Esbjerg, Denmark; 11 Naturalis Biodiversity Center, Leiden, The Netherlands; 12 Institute for Terrestrial and Aquatic Wildlife Research, University of Veterinary Medicine Hannover, Foundation, Büsum, Germany; Aristotle University of Thessaloniki, Greece

## Abstract

Ecological indicators for monitoring strategies are expected to combine three major characteristics: ecological significance, statistical credibility, and cost-effectiveness. Strategies based on stranding networks rank highly in cost-effectiveness, but their ecological significance and statistical credibility are disputed. Our present goal is to improve the value of stranding data as population indicator as part of monitoring strategies by constructing the spatial and temporal null hypothesis for strandings. The null hypothesis is defined as: small cetacean distribution and mortality are uniform in space and constant in time. We used a drift model to map stranding probabilities and predict stranding patterns of cetacean carcasses under H_0_ across the North Sea, the Channel and the Bay of Biscay, for the period 1990–2009. As the most common cetacean occurring in this area, we chose the harbour porpoise *Phocoena phocoena* for our modelling. The difference between these strandings expected under H_0_ and observed strandings is defined as the stranding anomaly. It constituted the stranding data series corrected for drift conditions. Seasonal decomposition of stranding anomaly suggested that drift conditions did not explain observed seasonal variations of porpoise strandings. Long-term stranding anomalies increased first in the southern North Sea, the Channel and Bay of Biscay coasts, and finally the eastern North Sea. The hypothesis of changes in porpoise distribution was consistent with local visual surveys, mostly SCANS surveys (1994 and 2005). This new indicator could be applied to cetacean populations across the world and more widely to marine megafauna.

## Introduction

Top predators have long been considered as conservation priorities [1]–[4]. The generally low resilience of these species results from their low fecundity and their position at the top of food webs and makes them more susceptible to many human-induced pressures (direct takes, competition with fisheries, by-catch, bio-accumulation of persistent contaminants). Because top predators rely on lower trophic levels for their food, their conservation implies a sustainable management of their prey and the preservation of ecosystem processes that determine the development of forage organisms. Finally, because most top predators have extensive home ranges, their conservation should envisage large subareas. Due to their often iconic nature, the presence of top predators can be a lever by which many less popular organisms can benefit from the protection afforded to the habitats shared with top predator ‘flagship’ species [2], [3], [5]–[8]. The need and efficacy of conservation plans must be assessed by implementing monitoring programmes.

Monitoring is defined as “the long term collection and analysis of repeated observations or measurements to evaluate changes in condition and progress toward meeting a management objective” [9]. The efficiency of a monitoring plan is based on three expected performances: ecological relevance, statistical credibility and cost-effectiveness [10], [11]. Nevertheless, collecting data often remains very expensive, putting management objectives at risk [11]. Indicators are therefore being used as a simplification of recorded parameters. Indicators are defined as measures established from verifiable data that include more information than data themselves do. As a low cost simplification of the monitored parameters, indicators allow communication between scientists and policy-makers [4], [12]–[14].

Cetaceans in Europe are protected by many international and national regulations (e.g. European Union Habitats Directive, Marine Strategy Framework Directive and Common Fisheries Policy; US Endangered Species Act and Marine Mammal Protection Act; the OSPAR Convention; ASCOBANS). Several parameters can be measured to provide relevant information on cetacean population status: absolute abundance, relative abundance, distribution, demographic parameters and health status. Most of these parameters (absolute and relative abundance and distribution) require extensive data to be collected at sea, generally at high costs. However, the efficiency of monitoring plans is also based on cost-effectiveness, and the development of low-cost indicators for assessing cetacean population status is of great interest. Hence, the present study was aimed to examine the potential of stranding data to provide indicators for cetacean populations. For scientific purposes, the word “stranding” is commonly used for either live or dead specimen [15]; in the present work, we will only be considering dead specimens washed ashore.

It is commonly acknowledged that stranding data are relatively inexpensive to collect, because they do not rely on the implementation of expensive field work conducted at sea (even if necropsies and other investigations and analyses on carcasses remain expensive). Therefore, many attempts for testing the value of strandings as a source of indicators of mortality at-sea have been made, mostly for seabirds [16], [17], sea otters [18], [19], sea turtles [20]–[22] and cetaceans [23]–[26]. In several European countries, marine mammal strandings have been recorded for decades. Stranding data held in national data-bases jointly constitute one of the largest datasets about cetaceans in European waters. Thanks to their fairly low running costs, national stranding schemes have developed in most European countries and cover extensive spatial (1000 s km coastline) and temporal (several decades) ranges that are consistent with the characteristics of cetacean populations (extensive population home ranges, low recovery rates). During the last 20 years cetacean stranding networks have been aimed at contributing monitoring strategies by collecting data on *inter-alia* spatio-temporal patterns of occurrence, cause of death, health status, ecological traits and population structure [27]–[32]. Nevertheless, the use of stranding data is often limited by the opportunistic nature of sampling and the difficulty to relate patterns and figures observed in strandings with processes affecting populations [33]. Nonetheless, the scientific use of strandings as a source of population indicators is encouraged by a variety of intergovernmental dispositions or recommendations (International Whaling Commission; various agreements under the Convention for Migratory Species; International Council for the Exploration of the Sea; OSPAR Convention; Marine Mammal Protection Act…). Therefore, it becomes particularly important to delineate their ecological significance and statistical credibility.

The harbour porpoise (*Phocoena phocoena*) is listed in many international conventions, directives and agreements (e.g. Conservation of Migratory Species of Wild Animals, EU Habitats and Species Directive, Protocol for Special Protected Areas and Biological Diversity, Convention on International Trade in Endangered Species of Wild Fauna and Flora, Agreement on the Conservation of Small Cetaceans of the Baltic, North East Atlantic and North Seas (ASCOBANS)) [34]. Monitoring harbour porpoise populations is requested by an increasing number of dispositions, including the ASCOBANS conservation plan in the North Sea and recovery plan in the Baltic Sea. Indeed, the harbour porpoise is impacted by anthropogenic disturbances, mostly fishery activities (competition and bycatch) [27], [35]–[40], organic pollutants and heavy metals contamination [28], [41]–[45] and recently the exponential growth of industrial activity at sea through the construction of offshore wind farms [46], [47]. The existence of pressing conservation issues and the broad distribution of the harbour porpoise in European waters prompted us to concentrate the present study on this species. The study area covered the north-eastern Atlantic waters of the Bay of Biscay and the North Sea, hence encompassing an extensive part of the species distribution in European waters.

Cetacean strandings follow a complex function of a biological component that is abundance and mortality rate, and a physical one that is drift processes, including carcass buoyancy and reporting conditions.

N_stranding_ ∼ N_individual_.mortality.buoyancy.drift.reporting.

Drift conditions being mostly driven by wind and tide are likely to introduce much noise in stranding data series. Here we explore how drift varies spatially and temporally and assess its contribution to variation in cetacean stranding numbers. By using the drift model MOTHY developed by MétéoFrance, the French meteorological agency, we propose to examine how harbour porpoise (and by extension any small cetacean) stranding should be distributed if variations were only due to drift conditions (abundance, mortality rate and reporting rate being set uniform and constant). As such, we built the null hypothesis (H_0_) of stranding records and made predictions of *inter-alia* long term stranding series and seasonal variations at various spatial scales across study area, against which observed stranding data, provided by six contiguous national stranding schemes (from north to south: UK, Denmark, Germany, The Netherlands, Belgium and France) can be statistically compared in a rigorous hypothesis testing procedure. The main goal of the current study is to improve the statistical credibility of stranding records as indicators for marine megafauna in a monitoring perspective at international scale. For the first time in Europe, administrative boundaries were pulled down to work at cetacean population scale.

## Materials and Methods

### 1-General Experiment Design

**Figure 1:**
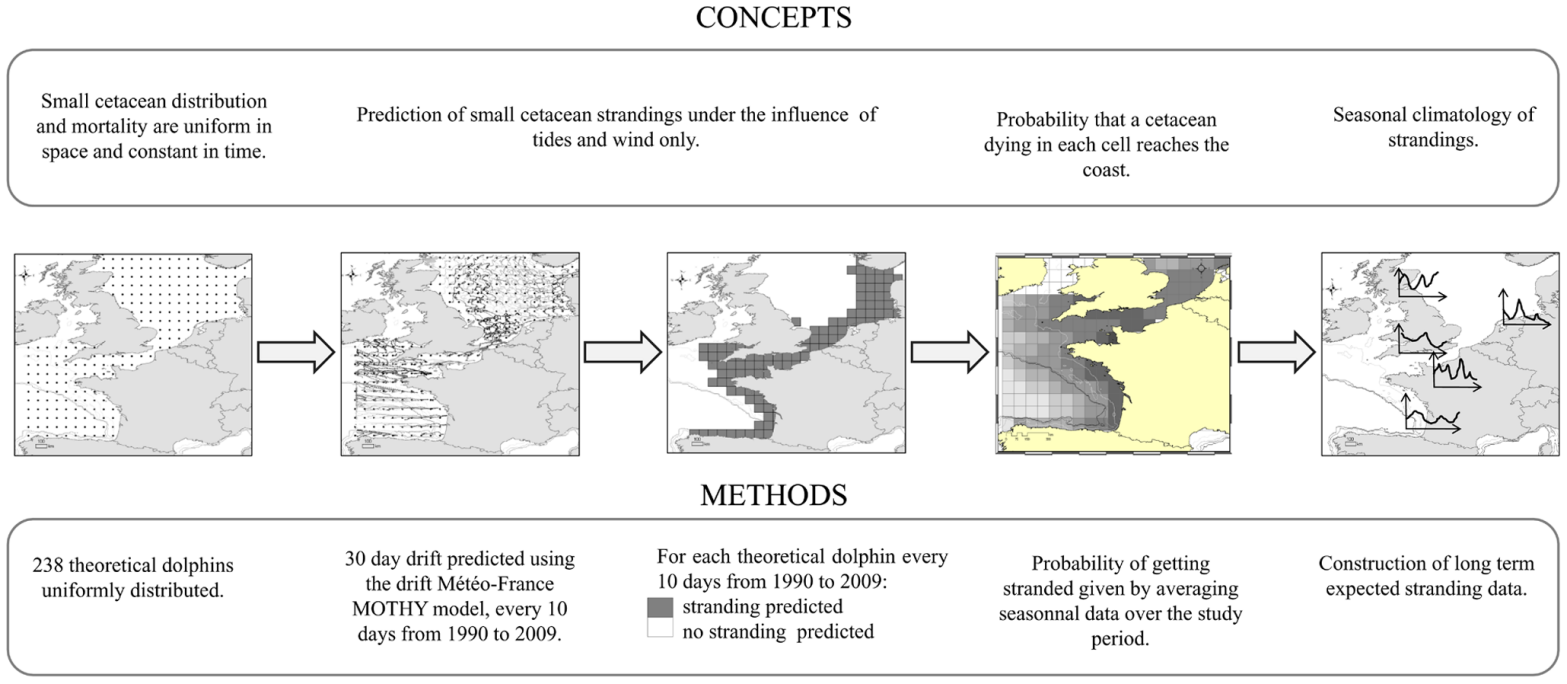
Theoretical scheme of the experiment.

The study area covered the Bay of Biscay, the Channel and the North Sea (8°50′W–10°00′E; 43°00′N–59°00′N).and ranged from 1990 to 2009. Small cetacean stranding time series and seasonal patterns calculated under the null hypothesis reflect stranding variations expected under the effect of tides and wind only, with the assumption that dead cetacean occur uniformly in time and space. They were constructed following four steps (figure 1).

Firstly, the hypothesis of spatial and temporal uniformity of dead harbour porpoises was represented by theoretical cetaceans uniformly distributed in a gridded map of the study area. Their drift was computed for 30 day every 10 days from 1990 to 2009, by using the drift prediction model MOTHY, and the value of 1 was attributed to its cell of origin if it was predicted to strand and 0 if not. Gridded maps of 0 and 1 were built and yearly, seasonally or monthly averaged to represent the probability that a cetacean dying in each cell reaches the coast and get stranded, under the influence of tides and wind only (*P_stranding_*). Finally, these simulations allowed 20-year-long strandings time series to be predicted under the null hypothesis. These predictions were compared to observed strandings along European coasts in order to highlight differences with the hypothesis of uniformity in abundance and distribution of dead cetaceans, and thus underline the biological component of harbour porpoise stranding records.

### 2-Definitions

We defined the probability of strandings (*P_stranding_*) as the probability that a cetacean dying at sea reaches the coast and gets stranded.

Expected strandings are strandings predicted under the hypothesis of spatial and temporal uniformity of dead cetaceans. They vary in time and space with drift conditions only.

Observed strandings are strandings collected by all European stranding networks operating across study area.

The difference between observed and expected strandings was named the anomaly of strandings. Positive (*vs* negative) anomalies suggest that more (*vs* less) strandings were observed than expected under the null hypothesis.

We concluded that there was a seasonal pattern (or seasonality), when monthly numbers of stranding events averaged over 20 years showed maximum (*vs.* minimum) figures during three or more consecutive months.

Long term data were used to describe the 20 year time series at either annual or monthly resolutions.

### 3-Construction of the Null Hypothesis

#### Drift prediction model MOTHY

The drift of cetacean carcasses was modelled with the drift prediction model MOTHY (*Modèle Océanique de Transport d’HYdrocarbures), initially developed by Météo-France* [48] to predict the drift of oil slicks and adapted later on to solid objects. MOTHY predicts trajectories of floating objects by calculating the vertical profile of currents and the wind effect on the emerged part of the object. MOTHY can be used forward (from drift start to landing point) or backward (from landing location to drift origin) [48].

The bathymetry used by MOTHY was compiled from data provided by SHOM (*Service Hydrographique et Océanographique de la Marine*) at a resolution of 0.08°.

Atmospheric data, provided by the European Centre for Medium-Range Weather Forecasts (ECMWF), combines forecast outputs and data assimilation processes. Tides are modelled using a purely hydrodynamic tidal model. Water velocity is generated by a coupling between a 2D hydrodynamic limited area ocean model and a 1D eddy viscosity model [48].

Object characteristics (thickness and buoyancy), date, starting location and duration of the drift are needed. These parameters were adapted to small cetaceans (size: 2 m; total thickness: 0.32 m; first (or last in the case of back-calculation) date and location documented on a case by case basis), and immersion rate was experimentally estimated at 90% [26].

#### Drift calculations

The hypothesis of spatial and temporal uniformity of dead porpoises at sea was represented by a grid of 238 theoretical small cetaceans uniformly distributed cells of 0.75° longitude and latitude and corrected by the projection distortion according a north-south gradient (figure 1).

Drifts were calculated for 30 days every 10 days, from 1990 to 2009. The 30 day threshold was chosen according to the decomposition status of carcasses and the change of immersion rate at this stage [26].

#### Stranding climatology

Gridded maps were constructed every 10 days from 1990 to 2009. For each cell, 1 was attributed if the theoretical cetacean dying in this cell was predicted to strand and 0 if not. A total of 720 maps were obtained. They were averaged over 20 years to construct climatology maps of stranding probability for small cetacean at various time frames, including month, season and year.

Finally, time series of expected strandings were constructed with a 10 day resolution, along the European coast divided in eight sub-areas (Bay of Biscay, western Channel, eastern Channel, south-western North Sea, north-western North Sea, south-eastern North Sea, mid-eastern North Sea and north-eastern North Sea). The number of stranded cetaceans expected per coastal kilometre per year was an indicator of the exposure of each stretch of coasts to small cetacean strandings under the null hypothesis; in this exercise coastline was measured in straight line along the main orientation of each sub-area.

### 4-Harbour Porpoise Stranding Data

Harbour porpoise stranding time series were compiled from six European countries: Denmark, Germany, the Netherlands, Belgium, United Kingdom and France. Date and location of strandings, as well as cause of death were collected. Live stranding events were not considered as the stranding location is not entirely determined by drift conditions but even by active swimming of animals.

#### United Kingdom stranding network

The stranding network in the United Kingdom is one of the oldest organisations in Europe that collects data on stranded cetaceans. The collaborative UK Cetacean Strandings Investigation Program (CSIP, www.ukstrandings.org) as it is now known is a consortium of partner organizations funded by Department of Environment, Food and Rural Affairs and the UK Devolved Administrations. Partner organizations are the Zoological Society of London, Scottish Agricultural College (Inverness), the Natural History Museum and Marine Environmental Monitoring. In Cornwall, strandings data is collected by the Cornwall Wildlife Trust Marine Strandings Network and necropsies are carried out by the Animal Health and Veterinary Laboratories Agency (Truro). The CSIP is collectively tasked with recording information on all cetaceans, marine turtles and basking sharks that strand around UK shores each year and with the routine investigation of causes of mortality through necropsy of suitable strandings. Experienced pathologists and biologists carry out systematic necropsies of selected stranded cetaceans following a standardized protocol.

#### Danish stranding network

The Danish stranding network is run by the Danish Nature Agency in collaboration with the Fisheries and Maritime Museum and the Zoological Museum, Natural History Museum of Denmark. Post mortems on stranded marine mammals are conducted by the National Veterinary Institute. The stranding network was founded in 1991 and relies on official personnel as well as reporting from the public.

#### German stranding network

The German stranding network at the North Sea coast was established in 1988/89 during the first Phocine Distemper Virus-Seal-die-off. National Park Rangers and “Seal hunters” (seals still belong the hunting law even as hunting was stopped in 1976) control the coastline regularity throughout the year so that a regular effort is secured. In Schleswig-Holstein marine mammal carcasses are collected and submitted for investigations.

#### Dutch stranding network

The Dutch strandings network consists of a consortium of a large number of organizations and volunteers. Coverage of the coast is very good along the south-western and western coasts of the country (approaching 100%) and on the westernmost Frisian island of Texel (coverage estimated 80%), but rather poor in the Wadden Sea and the remainder of the Frisian Islands, of which some are uninhabited. The central digital database is kept by Naturalis Biodiversity Center (formerly called the National Museum of Natural History *Naturalis*) in Leiden. Data and photographs are made visible on the internet (www.walvisstrandingen.nl).

#### Belgian stranding network

Strandings were collected in Belgium since 1970’s, but the dedicated and government supported network was established in 1990. It is organised and centralised by the Management Unit of the North Sea Mathematical Models (MUMM), department of the Royal Belgian Institute of Natural Sciences (RBINS). MUMM maintains, in cooperation with the University of Liège, a single database which can partly be consulted online.

#### French stranding network

The French stranding network is co-ordinated by the Joint Service Unit *PELAGIS*, UMS 3462, University of La Rochelle-CNRS, dedicated to monitoring marine mammal and seabird populations, as a continuation of the monitoring programmes formerly known as the *Centre de Recherche sur les Mammifères Marins* (CRMM). The network is constituted of around 260 trained volunteers distributed along the whole French coast who collect data according to a standardized observation and dissection protocol. The network was established in the early 1970’s and its organisation and procedures are considered unchanged since the mid 1980’s. Data are centralized into a single database held in La Rochelle.

### 5-Time Series Analysis

The anomaly of stranding time series was built as the difference between observed and expected harbour porpoise strandings time series. To do this the expected stranding time series was first calibrated with observed stranding time series in each large area in order to obtain equal cumulated numbers in both series.

Firstly, the difference between observed and expected strandings was tested by Wilcoxon test for non-parametric paired samples, for each large area both spatially and temporally. Secondly, the seasonality of the stranding anomaly was described over 20 years, with a correlogram produced by an autocorrelation function (ACF), using the software R [49], [50]. This analysis disentangles seasonal signal and trends and detects autocorrelations in time series at different lags. A lag corresponds to the temporal resolution of the time series, here one month. In this case, a year would be represented by 12 lags. The ACF analysis showed the degree of autocorrelation in time series at each lag (from 0 to 24 that is two years), and revealed the existence of seasonal signal in long term series.

Changes in stranding anomaly were detected using an algorithm for detecting breaks in time series, based on the F-statistics [51]. This algorithm detects structural changes in a linear regression by testing the regression coefficients and can be applied to time series.

### 6- Ethics Statement

This work reports on new results that have never been and are not being submitted elsewhere. This work was carried out in the respect of European regulation regarding the use of stranded dead cetacean for scientific and conservation purposes. The authors have therefore adhered to general guidelines for the ethical use of animals in research, the legal requirements in Europe. No living animals were used for this study, only dead cetaceans found stranded along European coasts by several organisations were considered. No samples were used for this study. The collect of dead stranded animals is delegated to regional or national organisms under the permission different institutions. In the United Kingdom, the Department of the Environment, Food and Rural Affairs is the authority to remove animals for post-mortem examination. The Royal Belgian Institute is appointed by law in Belgium; the same goes for the Netherlands where the Dutch law is the authority who has issued Naturalis the permission to collect stranded marine mammals. In Lower Saxony (Germany), the authority is either the National Park Authority Wadden Sea of Lower Saxony or the nature conservation authority of the county who grants a permit to collect dead cetaceans. In Schleswig Holstein (Germany), this is the State office for agriculture, environment and rural areas. In Denmark, the relevant authority is the Danish Nature Agency, which is under the Danish Ministry of the Environment. Finally in France, the Ministry for environment, sustainable development and ecology issues the permission to collect strandings to the Pelagis Observatory (University of La Rochelle).

## Results

### 1-Climatology of Small Cetacean Stranding Events in Northwest European Waters

#### Stranding probability (P stranding)

**Figure 2:**
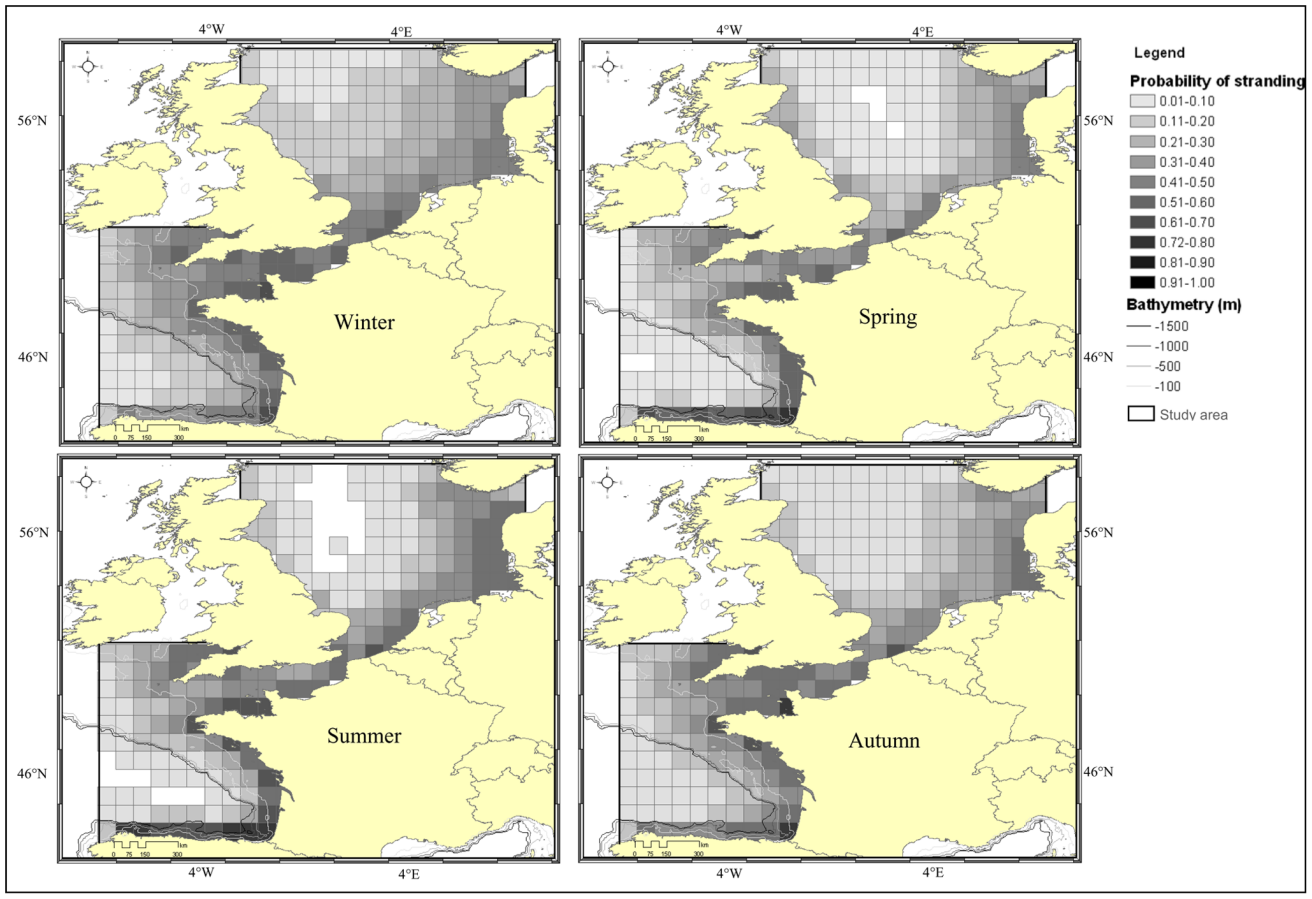
Seasonal maps of stranding probability in the study area from 1990 to 2009. The darker the colour the higher the probability that animals dying in the corresponding cell would reach the coast.

Maps of stranding probability were constructed, for each season averaged over 20 years (figure 2). Seasons were defined on the basis of the proportion of cells with *P_stranding_* = 0 (figure 2). December to March were gathered together into a winter season, because all cells were non-null. The spring season constituted of April to June, because cells with *P _stranding_* = 0 represented <10% of the study area. July and August presented a proportion of null cells >10% and constituted the summer season. Finally, months from September to November were pooled into an autumn season, because *P_stranding_* = 0 in less <5% of all cells.

The probability that cetaceans get stranded is always higher in coastal area.

The areas with high probabilities of stranding expand in winter and shrink in the summer as a result of seasonal variations in prevailing wind force. During winter months only 12.5% of the whole study area presented *P_stranding_* <0.1. Highest probabilities of strandings (>0.3) covered 63% of the study area, over the continental shelf of the Bay of Biscay, the English Channel and the eastern half of the North Sea. These areas retracted during spring months to 52% and a broad area of low stranding probability appeared in the central North Sea. In the summer months, around 10% of the study area showed *P_stranding_* = 0, suggesting that a cetacean dying in these areas would never get stranded and cells with *P_stranding_* >0.3 covered no more than 49% of the study area. These cells were observed over the continental shelf in the Bay of Biscay and in the western half of central North Sea. Autumn drift conditions showed *P_stranding_* >0.3 covering 57% of the study area.

#### Exposure of European coasts to small cetacean strandings

**Figure 3:**
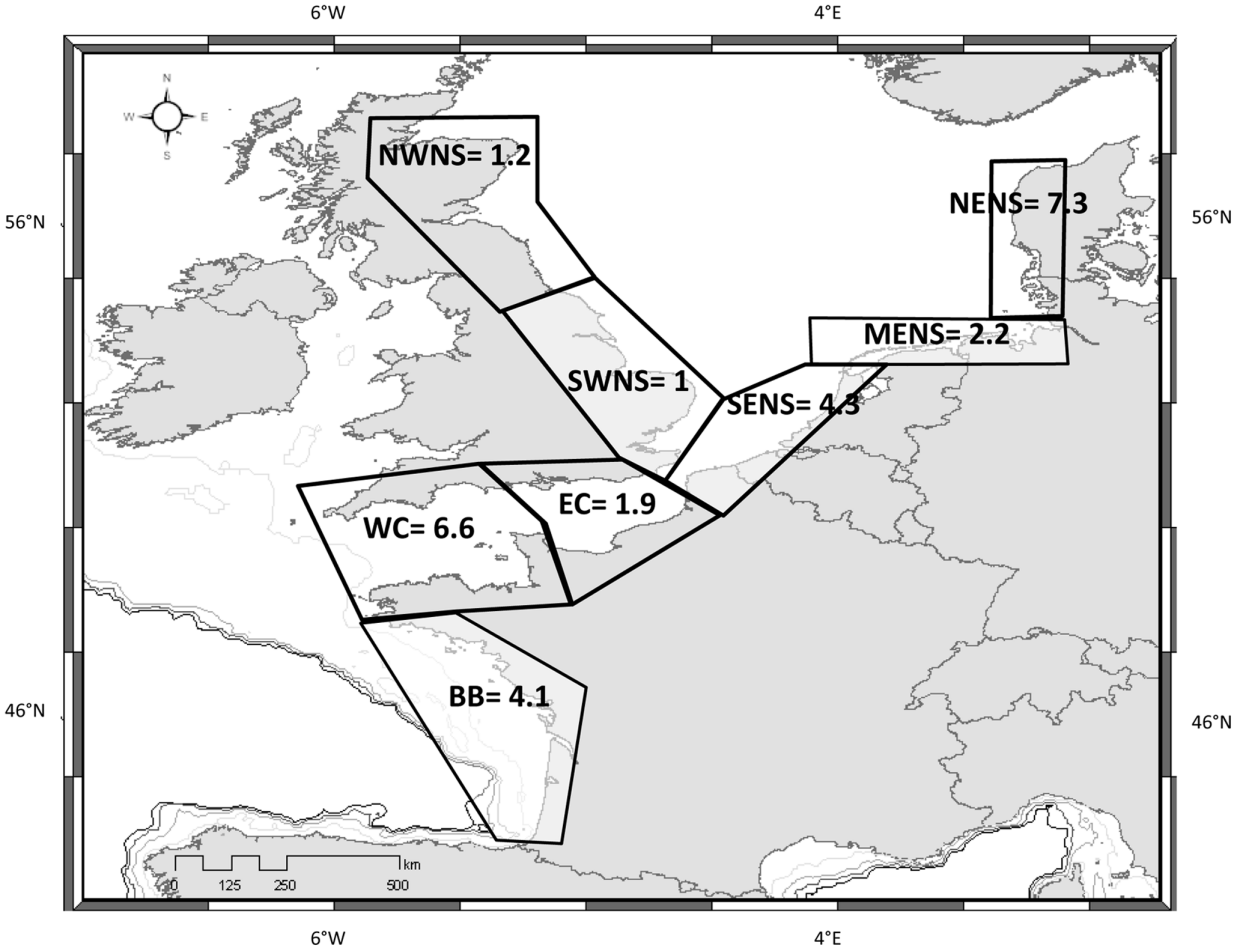
Relative numbers of expected strandings along the coasts of seven large subareas from 1990 to 2009 (stranding.km ^−1^.year^−1^). BB: Bay of Biscay, WC: western Channel, EC: eastern Channel, SWNS: south-western North Sea, NWNS: north-western North Sea, SENS: south-eastern North Sea, MENS: mid-eastern North Sea, NENS: north-eastern North Sea.

For each large sub-area, the number of expected strandings per coastal kilometre per year was calculated. To facilitate comparison between large subareas, an arbitrary value of 1 expected stranding.km^−1^.year^−1^ was given to the stretch of coast that showed the lowest number of expected strandings, in this case the south-western North Sea coast, and figures for the other sub-areas were obtained proportionally. Highest values were found in north-eastern North Sea and western Channel (7.3 and 6.6 stranding.km^−1^.year^−1^ respectively; figure 3). Lowest values were found in the north-western and south-western North Sea (1.2 and 1.0 stranding.km^−1^.year^−1^ respectively; figure 3).

#### Seasonal patterns of exposure to strandings

**Figure 4:**
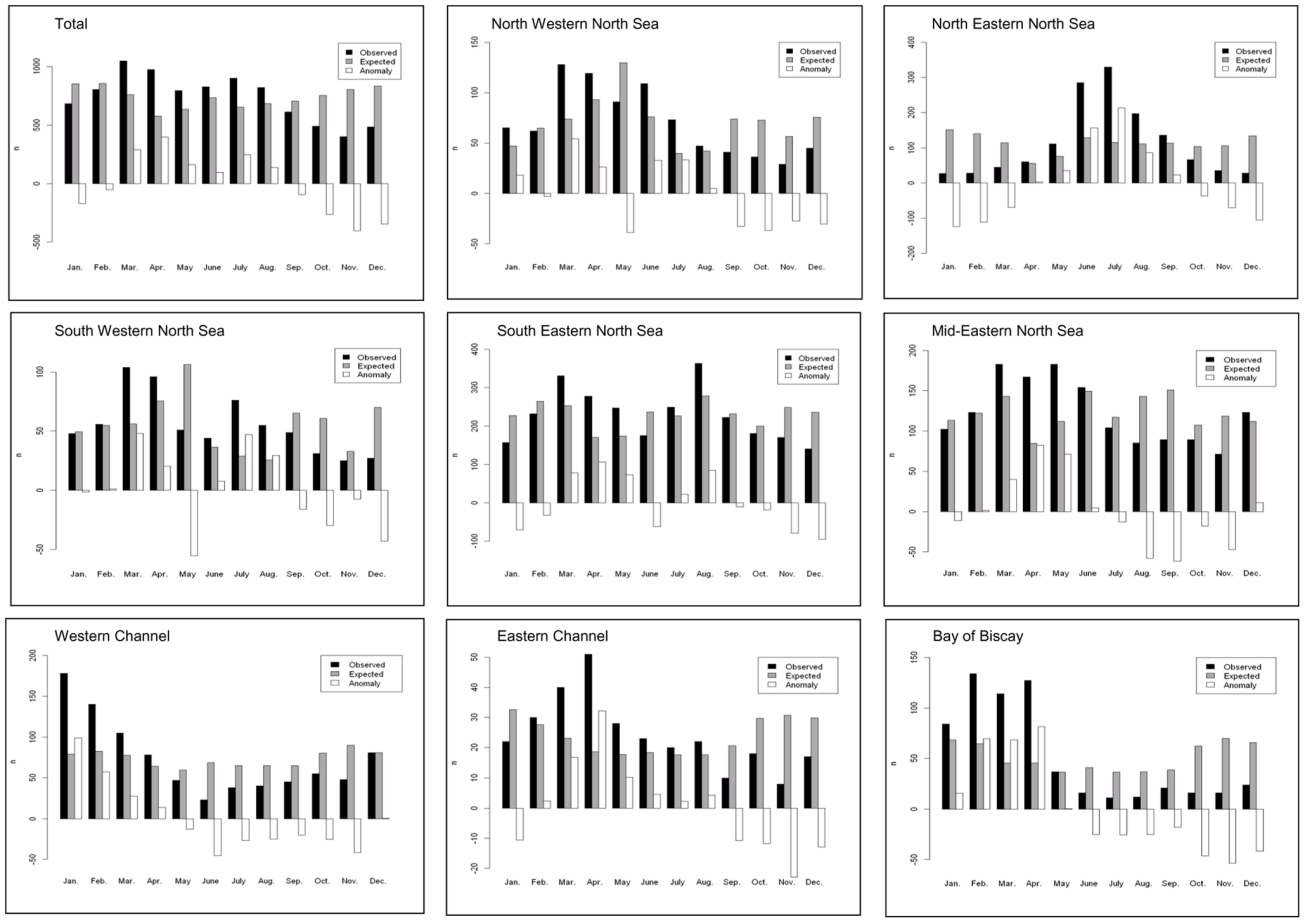
Average monthly distribution of observed strandings (black bars), expected strandings (grey bars) and stranding anomaly (white bars) (n) from 1990 to 2009.

Monthly numbers in expected stranding in numbers.km^−1^ were averaged over 20 years to detect seasonal patterns in expected time series (figure 4). Seasonality was detected in the Bay of Biscay and western Channel sub-areas. Maxima were observed between October and February and numbers decreased between May and August down to 34–50% of the highest numbers. In these three regions, coefficients of variations were very low (0.27 in the Bay of Biscay, 0.25 in eastern Channel and 0.13 in western Channel).

Seasonal patterns along western and eastern North Sea coasts were opposite, regardless of north-south divisions. In south-western and north-western North Sea, expected strandings increased in winter to reach a maximum in May being more irregular during the rest of the year. In south-eastern, mid-eastern and north-eastern North Sea, monthly expected strandings were irregular but minima were observed in April and May.

### 2-Harbour Porpoise Stranding Patterns in Northwest European Waters

#### Spatial patterns in observed harbour porpoise stranding data

**Figure 5:**
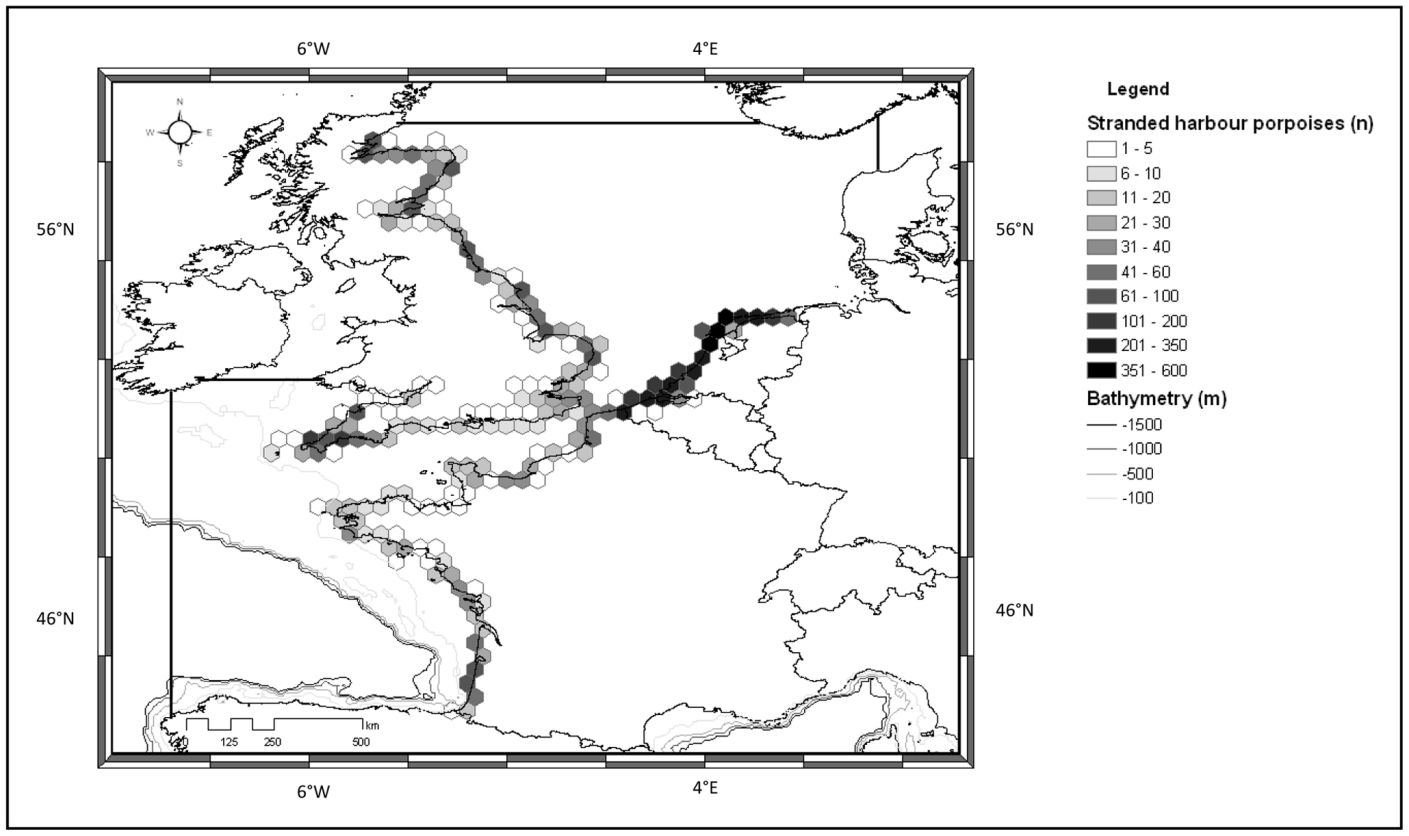
Harbour porpoise strandings collected by European stranding schemes (n) from 1990 to 2009.

A total of 10,038 stranded harbour porpoises were used from across the whole study area (figure 5): 2,534 in the north-eastern North Sea, 1,473 in the mid-eastern North Sea, 2,745 in the south-eastern North Sea, 845 in the north-western North Sea, 662 in south-western North Sea, 289 in eastern Channel, 878 in western Channel and 607 in the Bay of Biscay, over the study period 1990–2009. The average frequency of stranding events was 0.12 harbour porpoise stranded.km^−1^.year^−1^, coastline being measured in straight line along the main orientation of the sub-areas.

The highest frequency of stranded harbour porpoises was observed along the south-eastern North Sea coast, where it reached 0.4 strandings.km^−1^.year^−1^. High frequencies were observed along the mid-eastern North Sea with 0.28 strandings.km^−1^.year^−1^ and 0.32 along the north-eastern North Sea. North-western parts of the North Sea and the Channel showed lower frequencies with 0.09 strandings.km^−1^.year^−1^. Finally the lowest frequencies were found in the south-western North Sea (0.06 stranding.km^−1^.year^−1^), in the Bay of Biscay (0.05 stranding.km^−1^.year^−1^) and along the eastern Channel coast (0.03 stranding.km^−1^.year^−1^). There appears to be good consistency in strandings frequencies across national boundaries, suggesting that possible differences in reporting effort between countries would not be a major source of heterogeneity.

#### Long term trends in observed harbour porpoise stranding data

**Figure 6:**
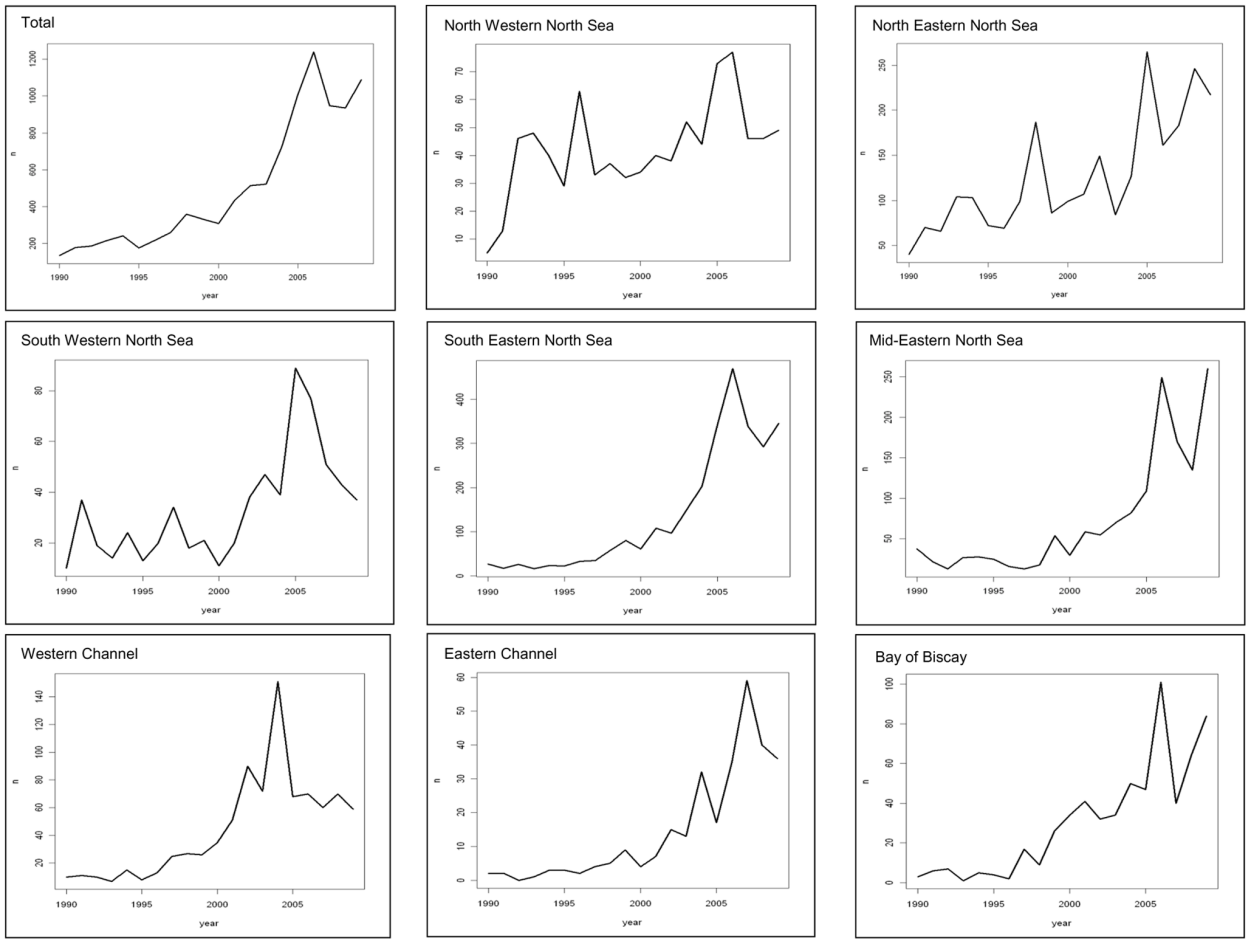
Annual numbers of observed harbour porpoise strandings (n) from 1990 to 2009.

Over the whole study area, porpoise strandings showed a strong increase over the study period, mostly since 2000 (figure 6). The maximum of 1 240 porpoises was reached in 2006. In the last 3 years, numbers showed a slight decrease. In the north-western North Sea, porpoise strandings were increasingly observed since 1990, with a maximum of 77 strandings reached in 2006. Along the south-western North Sea coast, numbers highly increased from 2000 onwards. In the north-eastern North Sea, strandings were increasingly reported since 1990, with maximum number recorded in 2005 (265 strandings). In the mid-eastern and south-eastern North Sea, averages of 15 and 35 strandings.year^−1^ respectively were observed in the 1990’s. Since 2000, higher numbers were recorded. Along the eastern Channel, less than 10 strandings.year^−1^ were observed until 2001.Since 2002, numbers increased to 59 porpoises stranded in 2007. Around 10 porpoises were found stranded in the western Channel every year between 1990 and 1996, before increasing to a maximum of 151 strandings in 2004. Since 2005, numbers decreased. Along the Bay of Biscay, an average of 4 porpoises.year^−1^ was observed stranded from 1990–1996. From 1997 on, numbers increased to 101 recorded in 2006 and lower numbers were observed since then (around 63 strandings.year^−1^).

#### Seasonal patterns in observed harbour porpoise stranding data

Harbour porpoise stranding numbers were summed by month to highlight seasonal distribution of strandings across the study area: as many as 53% of porpoise strandings were observed between March and July (figure 4). Except in north-eastern North Sea, highest numbers were observed between March and May (twice higher than during the rest of the year). During spring or summer, high stranding numbers were recorded in one month. In the north-eastern North Sea, strandings recorded in June and July were 15 times higher than in winter. The coefficient of variations was one of the highest in the study area (CV = 0.94). In the Channel, seasonal patterns showed highest values from January to April. In winter, the maximum value was observed in January in the western part and decreased regularly until June, whereas numbers increased from January to April in the eastern Channel. Along the Bay of Biscay coasts, highest porpoise numbers were observed from January to April, and very few were recorded the rest of the year. The high variations between winter and summer were confirmed by the highest coefficient of variation (CV = 0.96).

### 3-Stranding Anomalies

#### Spatial comparisons

We compared expected strandings.km^−1^.year^−1^ in the 8 large European regions to observed strandings.km^−1^.year^−1^ collected by stranding networks in these subareas and found that difference was not statistically significant (*P* = 0.480).

#### Long term trends

At the European scale, the difference between observed and expected harbour porpoise strandings was significant (*P* = 0.021). This result was found in all regions (*P*<10^−3^ in each case) except along the south-western and north-western North Sea coasts (*P* = 0.379 and 0.199, respectively).

**Figure 7:**
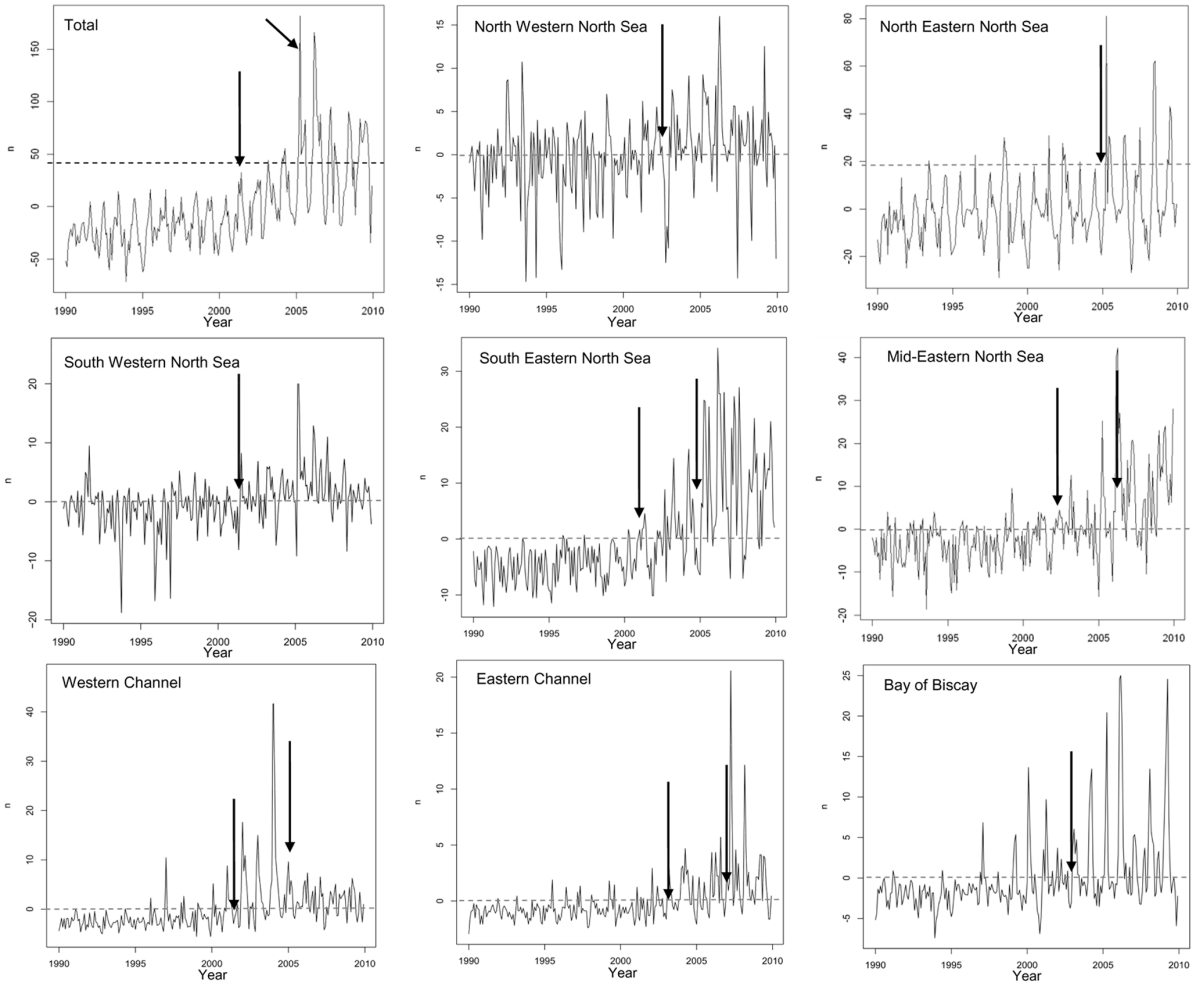
Long term harbour porpoise stranding anomalies in large subareas (n) from 1990 to 2009. Black arrows represent detected breakpoints in time series.

At a European scale, stranding anomaly showed a regular increase and two breakpoints were detected in 2001 and 2005 (figure 7). Trends in anomalies became positive after the first breakpoint and a strong increase started with the second breakpoint.

The Bay of Biscay, the Channel, the mid-eastern and the south-eastern North Sea showed negative anomaly from 1990 to 2000 that became positive thereafter. Since 2005, these anomalies became strongly positive and showing that there were much more porpoises observed stranded than expected under the null hypothesis. Along the western Channel coast, high positive anomalies were observed during a short period from 2002 to 2005. Since 2005, these values became more regular and closer to 0. The north-western, north-eastern and south-western North Sea coast showed different profiles and anomalies were steadier over the study period. Occasional high stranding anomalies were recorded in those areas. The decrease in stranding anomalies observed in recent years for several regions was not identified as a breakpoint by the analysis, possibly because not enough years after the apparent start of this putative new trend are available to date.

#### Seasonal patterns

**Figure 8:**
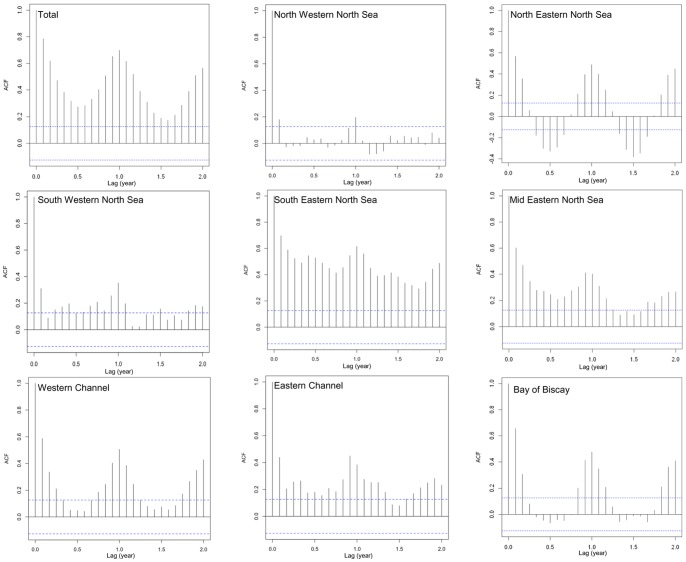
Correlograms of harbour porpoise stranding anomaly in large subareas from 1990 to 2009. If autocorrelation falls outside the dotted lines, it is considered to be significant 0 at a 5% probability level.

Correlogram of the stranding anomaly at European level showed a slow decrease with time (figure 8). The maximum correlation was found at a time-lag of 12 months, reflecting a positive linear relationship between variables separated by 1 year, highlighting the strong seasonal component of stranding data. The same general pattern was also observed along the south-eastern and mid-eastern North Sea coasts.

Very slight trends were detected in the Channel, as shown by the autocorrelations always >0, irrespective of time lag. Nevertheless a strong seasonality was detected with a 12-months period. Furthermore, the Bay of Biscay and north-eastern North Sea showed a negative correlation focused on 6-months periodicity, explained by a strong seasonal pattern and the lack of detectable trend in the time series. Finally, the north-western and south-western North Sea coast did suggest neither strong seasonal pattern nor trend, except a positive autocorrelation at lag 1 year, suggesting a slight 12-months pattern.

In the whole study area, the monthly decomposition of stranding anomalies showed that they were highest in April and July, and lowest from September to February (figure 4). A strong seasonality was observed in both Channel regions and in the Bay of Biscay, with positive anomalies in winter and negative during the rest of the year. The strongest seasonality was observed in north-eastern North Sea, with high positive anomaly calculated from June to August. In all other regions, this scheme was not so strong. Even if positive anomalies were still observed in late winter and spring, they remained irregular, with irregular changes in anomaly sign. The differences between observed and expected stranding seasonality were significant in the western Channel (*P* = 0.019), the Bay of Biscay (*P* = 0.019) and the north-eastern North Sea (*P*<10^−3^). In eastern Channel and in the rest of the North Sea, these differences were not significant (*P*>0.785).

## Discussion

### 1-General

This work is an entirely novel approach to analysing and interpreting small cetacean stranding data that is aimed at taking drift, the major confounding factor involved in the stranding process, into account when analysing stranding data sets. Firstly we constructed stranding time series expected under the assumption that dead small cetacean be uniformly distributed in time and space and therefore that spatio-temporal patterns in stranding frequency would entirely result from drift conditions. The exposure to strandings on the coast of 8 large European subareas was shown to vary by a factor of 1 to 7 depending on the orientation of the coast relative to prevailing winds. Secondly, expected small cetacean stranding time series and seasonal patterns were compared to observed harbour porpoise stranding data on the assumption that any deviation from the null hypothesis would help disentangle the complexity of observed stranding records and express their biological component, *i.e.* spatio-temporal patterns in combined harbour porpoise density and mortality.

Two important aspects of this approach are its large spatial scale (from the northern North Sea to the southern Bay of Biscay) and long temporal span (20 years). Uncertainty in carcasses drift modelling was estimated at a few 10 s of kilometres [26], well below the sizes of study area and sub-areas sizes. In addition, the size of the study area encompasses a large proportion of the harbour porpoise population distribution in north-western Europe and a scale at which massive changes in distribution were shown between 1994 and 2006 by the SCANS and SCANS II surveys [52], [53]. Furthermore, genetic analyses suggested that harbour porpoises from Bay of Biscay to the North Sea may constitute a single continuous system under isolation by distance [54]. The long duration of the analyses potentially allows changes in trends to be differentiated from higher frequency noise in stranding data. To cover this large geographical scope and temporal span we have lumped together data sets from seven distinct national stranding schemes. Although this is an obvious strength of the study, it also introduces a source of heterogeneity that is difficult to assess as a result of the specific history and management of each of these schemes and the levels a public awareness on these issues (with is central in the reporting process) that have evolved at different rates between countries (possibly also within countries).

The determination of 8 large subareas according to coast-line orientations rather than European boundaries smoothed stranding patterns collected at country scale, but is considered to be much more relevant on an ecological point of view than statistics analysed on a national basis. Nevertheless, increased porpoise strandings observed since 2000 along the Dutch, Belgian and northern French coasts [55], [56] are consistent with changes detected in the regions created for the present study. Increased strandings along western North Sea and Channel were also consistent with trends in stranding numbers collected along UK coasts since late 1990’s [38]. Seasonal patterns in stranding data analysis carried out at national level may be slightly different to the patterns observed in the present work, but the predominant seasonality with maxima in winter months remained strong in eastern North Sea, Channel and Bay of Biscay [38], [56]. Both stranding maxima reached in March and August along Belgian and Dutch coasts were identified in our south-eastern North Sea area [55]. The strong seasonality focused on summer months described along the north-eastern North Sea coasts were consistent with German stranding data [30].

The main tool used in this study was the drift prediction model MOTHY. MOTHY characteristics imposed a limitation to the number of theoretical cetaceans (238) which drift could be modelled at any single date and therefore constrained the resolution of the grid to 0.75° in latitude and longitude. Given the resolutions used to model distributions of top marine predators, their prey and human activities (*e.g.* ICES statistical grid cells) the resolution of the present work is largely consistent with most other relevant source of information. Another constraint of MOTHY relates to its geographical extent and justifies why Spanish and Irish data cannot be added.

The null hypothesis is used in ecology to construct a situation where nothing happens and to test the effect of several controlled parameters [57]. This hypothesis is used to disentangle the part of process appeared at random [58]. In ecology, one example among others was to use it to construct theoretical fish communities and test one by one several parameters [59]. This work is the first attempt to use a similar approach in the analysis of cetacean stranding data, in the aim of disentangling biological processes from drift-related processes.

### 2-Drift Conditions in Europe

The monthly climatology of stranding probabilities in European waters showed a clear seasonal pattern as a result of the strong difference observed between winter and summer drift conditions. Cells located in coastal areas provided strandings year round, as exemplified in the Channel. Conversely, cells located further offshore in the Bay of Biscay and in the western central North Sea could seasonally be too far away to allow any carcass originating from these cells to reach a coast within 30 days. Understanding how the probability of stranding varies seasonally and spatially is essential to appreciate the geographical representativeness of data collected from stranded cetaceans. Here, highest probabilities to get stranded appear close to the coasts in the summer and extend over the shelf in the winter.

Seasonal wind climatology explains the seasonality in stranding probability and expected strandings but only partially drives seasonal stranding patterns. Similar patterns were found along the Bay of Biscay and the Channel coasts with maxima expected in winter and minima in summer, but the amplitude of the seasonal patterns was much larger in observed stranding data than in expected stranding data. Opposite patterns are observed between western (maxima in May) and eastern (minima in April-May) coast of the North Sea. In north-western Europe, strongest wind systems are observed in winter, from October to February [60], [61]. Main wind orientation is from the north-west in the Bay of Biscay and west-south-west in the Channel and the North Sea [60], [61].

### 3-Interpretation of Harbour Porpoise Stranding Time Series

Positive (*vs*. negative) standing anomalies suggested that observed stranding numbers were higher (*vs*. lower) than expected. In other words, departures from predictions made under the null hypothesis (uniformity of distribution in space and time) reveal the spatio-temporal patterns of the biological components of harbour porpoise stranding records.

With the exception of the western North Sea, long term stranding anomalies were always significantly different from 0, indicating that drift alone cannot explain inter and intra-annual variations in harbour porpoise stranding frequency. On average, expected minima and maxima ranged from 1 to 3, whereas observed numbers ranged from 1 to 10 and stranding anomalies ranged from 1 to 500. Seasonality analyses (correlograms and Wilcoxon tests) showed that in the Bay of Biscay and western Channel, the seasonal pattern was predominant and did not explain by drift conditions. In eastern Channel and south to mid-eastern North Sea, the long term trends was predominant and the seasonal signal was partially explained by drift conditions. In western North Sea, a slight seasonal pattern was described, but no trend was detected in stranding anomaly.

These results suggested that dead harbour porpoise numbers increased since 1990 and were observed during the whole year. These results were consistent with several previous studies based on at-sea sighting analysis, suggesting that harbour porpoises were observed year-round and increased in the past 10 years mostly in North Sea [30], [62]–[65]. The seasonality detected in north-eastern North Sea can be explained by a seasonal movements and an increase of porpoise abundance along these coasts. Dedicated surveys showed an increase of porpoise encounter rate in summer along German and Danish coasts [30], [47]. Additionally, summer is the porpoise calving period and calves occurrence remain high in stranded porpoise time series [30], [47]. In the Bay of Biscay and western Channel, seasonality was the predominant signal. Only winter maxima increased since 2000, whereas the difference was always negative in the summer for the whole 20 years long study period. This could be explained by a change in harbour porpoise distribution in the summer. The use of platform-of-opportunity data collected mostly in the summer showed that porpoises are observed in the western Channel and very few sightings are recorded in the Bay of Biscay proper [66], [67].

Finally, south-western and north-western North Sea coasts presented more similarity with the null hypothesis than any other region in the study area. Observed and expected time series were not significantly different and this difference is quite randomly distributed around 0 across the study period. In all other regions, observed porpoise strandings were lower than expected under the null hypothesis before 2000 and higher thereafter. This would suggest that harbour porpoises were not uniformly distributed in time and space in European waters since 1990, except along the western North Sea coasts. In other words changes occurred in abundance and/or mortality of harbour porpoises in north-western European waters. This conclusion was consistent with the hypothesis suggested after the SCANS and SCANS-II surveys carried out in 1994 and 2005 [52], [53]. These surveys suggested a southern shift in the harbour porpoise distribution rather than a change in their abundance. The difference in time series calculated in the study area suggested an increase of dead porpoises since 2000. The change in the sign of stranding anomalies could be due to a change in abundance of dead porpoises in our calculation area.

In 1994 (at the time of the first SCANS survey) abundance and mortality of porpoises in northern North Sea (west and east) seemed to be fairly stable and close to the null hypothesis and were lower in the rest of study area, as suggested by generally negative stranding anomalies in all regions except northern North Sea. These results were in line with SCANS results, with highest densities of porpoises observed in the northern North Sea [52]. Since 2000, increasing dead porpoise numbers (increasing stranding anomalies) were observed first in the south-eastern and south-western North Sea, then in the western Channel, the south-eastern and mid-eastern North Sea, the Bay of Biscay and finally the eastern Channel as shown by breakpoint analysis. In 2005 (second SCANS survey), high stranding anomalies were observed in Bay of Biscay, western Channel and all areas of the eastern North Sea, whereas lower numbers were observed in the eastern Channel and the south-western North Sea. Stranding anomalies highlighted changes in porpoise abundance and/or mortality. Increases of these anomalies can suggest either an increase in porpoise abundance in the area or an increase in mortality rate. The pattern in stranding anomaly detected gradually during the 2000’s along the North Sea, the Channel and the Bay of Biscay would be more consistent with a movement of the population. Therefore stranding anomaly increases could be explained by an increase of porpoises at sea in these areas. These results were very consistent with SCANS II results, even if no harbour porpoises were observed in the Bay of Biscay during this survey [53]. This can be explained by the low encounter rate observed in summer in the Bay of Biscay [66], [67]; probably due to a strong seasonal pattern in distribution or habitat use in the Bay of Biscay. Moreover the efficiency improvement of stranding networks in some areas in the early 1990’s would suggest that changes in stranding anomaly would probably not be wholly biological in origin. Nevertheless, the magnitude of increase cannot be entirely explained by changes in reporting effort because most European stranding networks operate efficiently since decades.

Increase in harbour porpoise stranding anomaly occurring earlier in the western Channel than in the eastern Channel would suggest that the southward movement of animals would have occurred along both sides of the British Isles.

### 4-Strandings as a Monitoring Tool

The comparison and the relevance of results obtained in this study compared to sighting surveys conducted in the North Sea and adjacent waters considerably improves the interest of using strandings as a monitoring tool. The analysis of stranding anomalies rather than raw data cleared the stranding signal from the effect of drift conditions. The link between strandings and cetacean at sea became clearer and simplified the understanding of stranding time series. Dedicated surveys provided snap-shot pictures of small cetacean distribution and absolute abundance at decadal interval. The monitoring of strandings and the analysis of stranding anomalies would provide continuous distribution and relative abundance information, cleared from biases related to variations in drift condition. The improvement of the knowledge of the relationship between strandings and cetaceans at sea constitutes a major step in the use of strandings as indicators of at-sea populations. Monitoring stranding data could provide maps of the likely origin of porpoises at sea, to identify high mortality areas and incidences with human activities [26]. Stranding data will never provide abundance estimates, but the cost of dedicated surveys at European scale is too high to provide cetacean monitoring data. The combined use of both tools would be relevant in the development of an efficient monitoring strategy, notably in context of the Conservation Plan for harbour porpoises in the North Sea, carried out by ASCOBANS.

Finally, the use of stranding anomaly as an indicator of cetacean mortality could become an additional efficient tool for environmental watch and to detect variations in strandings according to changes of abundance or mortality of cetaceans at sea. For the first time, the integration of European data will allow to look at biological phenomenon rather than national variations. Cause of death could be identified by carcass examination and stranding anomalies could be calculated for each cause of death. It would provide relevant information on spatial and temporal variations in death causes, improve the efficiency of stranding monitoring and allow early decision making in case of unusual mortality events.

### 5-Conclusion

It is commonly admitted that stranding schemes provide low-cost indicators and yield reasonable data on the frequency of occurrence of species in the regions they cover [23], [24], [68]. Thus, strandings provide similar rank-order relative abundances as live surveys [23], [24]. Consequently, stranding data could provide relevant low-cost information on mortality areas at sea [26], relative densities, distribution, specific richness and numbers cleared from drift variations as well. This study constitutes a significant improvement in stranding data statistical credibility in the context of monitoring population and providing worldwide indicators for cetaceans and more widely for marine megafauna like sea turtles or seabirds. The construction of new indicators for wildlife monitoring strategies is still an issue of major concern in conservation biology, in particular in the context of the ever increasing number of proposed Marine Protected Areas.
