# Correction: The Stranding Anomaly as Population Indicator: The Case of Harbour Porpoise *Phocoena phocoena* in North-Western Europe

**DOI:** 10.1371/annotation/1ce2f54e-1eea-4dba-8742-05db1017b44f

**Published:** 2014-01-03

**Authors:** Helene Peltier, Hans J. Baagøe, Kees C. J. Camphuysen, Richard Czeck, Willy Dabin, Pierre Daniel, Rob Deaville, Jan Haelters, Thierry Jauniaux, Lasse F. Jensen, Paul D. Jepson, Guido O. Keijl, Ursula Siebert, Olivier Van Canneyt, Vincent Ridoux

Figure 5 was mapped without the dataset in entirety. Please see the corrected Figure 5 here: http://plosone.org/corrections/pone.0062180.g005.cn.tif

